# Verrucous Carcinoma of the Larynx Presenting as a Hairy Whitish Tumor

**DOI:** 10.1155/DTE.3.249

**Published:** 1997

**Authors:** Masahiro Kawaida, Hiroyuki Fukuda, Naoyuki Kohno

**Affiliations:** 1 Department of Otolaryngology Tokyo Metropolitan Ohtsuka Hospital 2-8-1, Minamiohtsuka Toshima-ku Tokyo 170 Japan; 2 Department of Otolaryngology Keio University School of Medicine 35 Shinanomachi Shinjuku-ku Tokyo 160 Japan; 3 Department of Otolaryngology Juntendo University School of Medicine 2-1-1, Hongo Bunkyo-ku Tokyo 113 Japan

## Abstract

A patient was encountered with verrucous carcinoma of the larynx that presented as a hairy whitish tumor. There was a recurrence because simple excision with forceps by endolaryngeal microsurgery was performed in the first operation. However, in the second operation endolaryngeal microscopic laser surgery using a direct laryngoscope was performed and followed by adjuvant chemotherapy with oral UFT, a combination of uracil and tegafur in a molar ratio of 4:1. The patient’s course has been favorable to date. The case is reported in this paper and discussed from the viewpoint of diagnosis and treatment of this neoplasm.


*Diagnostic and Therapeutic Endoscopy*, 1997, Vol. 3, pp. 249-254
Reprints available directly from the publisher
Photocopying permitted by license only

© 1997 OPA (Overseas Publishers Association)
Amsterdam B.V. Published in The Netherlands
by Harwood Academic Publishers
Printed in Singapore

### Verrucous Carcinoma of the Larynx Presenting as a Hairy Whitish Tumor

MASAHIRO KAWAIDA[1*], HIROYUKI FUKUDA[2] and NAOYUKI KOHNO[3]

[1]*Department of Otolaryngology, Tokyo Metropolitan Ohtsuka Hospital, 2-8-1, Minamiohtsuka, Toshima-ku, Tokyo 170, Japan*
[2]*Department of Otolaryngology, Keio University School of Medicine, 35 Shinanomachi, Shinjuku-ku, Tokyo 160, Japan*
[3]*Department of Otolaryngology, Juntendo University School of Medicine, 2-1-1, Hongo, Bunkyo-ku, Tokyo 113, Japan*

*(Received 27 August 1996; in final form 19 November 1996)*

**A patient was encountered with verrucous carcinoma of the larynx that presented as a hairy whitish tumor. There was a recurrence because simple excision with forceps by endolaryngeal microsurgery was performed in the first operation. However, in the second operation endolaryngeal microscopic laser surgery using a direct laryngoscope was performed and followed by adjuvant chemotherapy with oral UFT, a combination of uracil and tegafur in a molar ratio of 4:1. The patient's course has been favorable to date. The case is reported in this paper and discussed from the viewpoint of diagnosis and treatment of this neoplasm.**

*Keywords:* Verrucous carcinoma, laryngeal cancer, squamous cell carcinoma, human papillomavirus, UFT

### INTRODUCTION

Verrucous carcinoma is a clinical variant of well-differentiated squamous cell carcinoma and has a relatively low grade of malignancy. It has a predilection for the oral cavity, and it is relatively rare for it to arise in the larynx. A patient was encountered with this neoplasm arising in the membranous portion of the vocal fold that presented as a hairy whitish tumor. There was a recurrence because simple excision with forceps by endolaryngeal microsurgery was performed at the first operation. In the second operation, however, endolaryngeal microscopic laser surgery using a direct laryngoscope was performed and followed by adjuvant chemotherapy with oral UFT, a combination of uracil and tegafur in a molar ratio of 4:1. The patient's course has been favorable to date. This case is reported below and discussed from the viewpoint of diagnosis and treatment of this neoplasm.

* Corresponding Author: Dr. Masahiro Kawaida Tel.: +81-3-3941-3211, Fax: +81-3-3941-9557

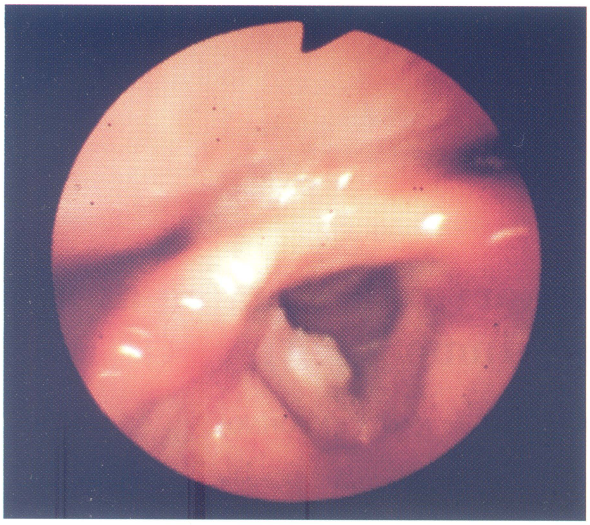

FIGURE 1 Flexible laryngofiberscopic findings at the time of the initial examination. A whitish exophytic lesion is observed extending from the center to the posterior of the membranous portion of the right vocal fold.

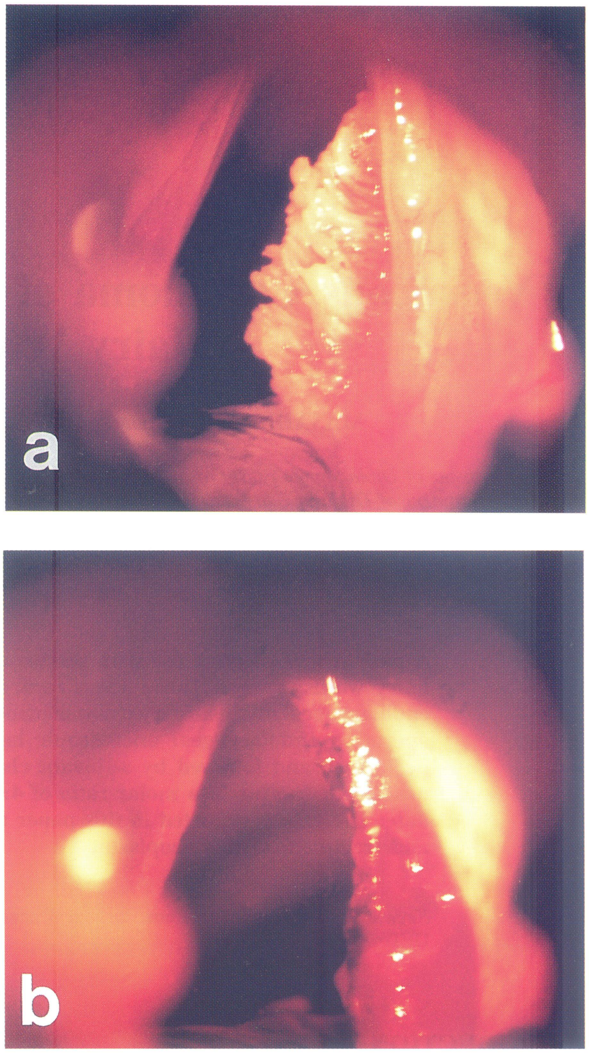

FIGURE 2 Laryngeal findings at the time of the first operation. a: (Before excision), A brush-like hairy whitish lesion is observed extending from the center to the posterior of the membranous portion of the right vocal fold. b: (After excision), The lesion alone has been excised from the surface of the vocal fold with forceps.

### CASE REPORT

### Clinical Course

The patient was a 55-year-old male who complained of hoarseness that he had first noted 6 months previously. In October 1993, he was examined at Tokyo Metropolitan Ohtsuka Hospital. His past history and familial history were unremarkable. He had a 35-year history of smoking 30 cigarettes a day. Flexible laryngofiberscopic examination revealed a whitish exophytic lesion extending from the center to the posterior of the membranous portion of the right vocal fold (Fig. 1). Inspection of the nasal cavity, oral cavity and pharynx failed to reveal any abnormal findings, and no cervical lymph nodes were palpable. The patient was admitted to our hospital in November 1993. Endolaryngeal microscopic examination and excisional biopsy using a direct laryngoscope were performed under inhalation anesthesia by endotrecheal intubation, and a brush-like hairy whitish lesion with exophytic outgrowth was observed extending from the center to the posterior of the membranous portion of the right vocal fold (Fig. 2a). The lesion alone was removed from the surface of the vocal fold with forceps (Fig. 2b). A histopathological diagnosis of hyperkeratosis was reported by the pathologist. The patient's course was therefore followed on an outpatient basis.

However, because a recurrence was detected and the lesion gradually grew larger beginning around August 1994, the patient was readmitted, and endolaryngeal

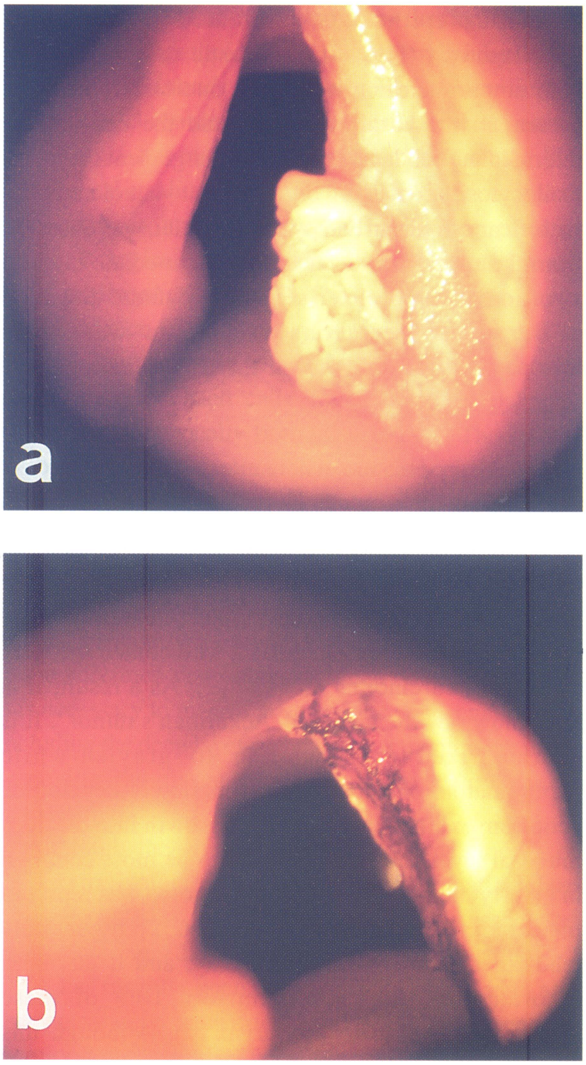

FIGURE 3 Laryngeal findings at the time of the second operation. a: (Before resection), A whitish lesion is observed in the membranous portion of the right vocal fold. b: (After resection), The lesion has been completely resected, and wide vaporization, including the surrounding normal mucous membrane, has been performed with a carbon dioxide laser.

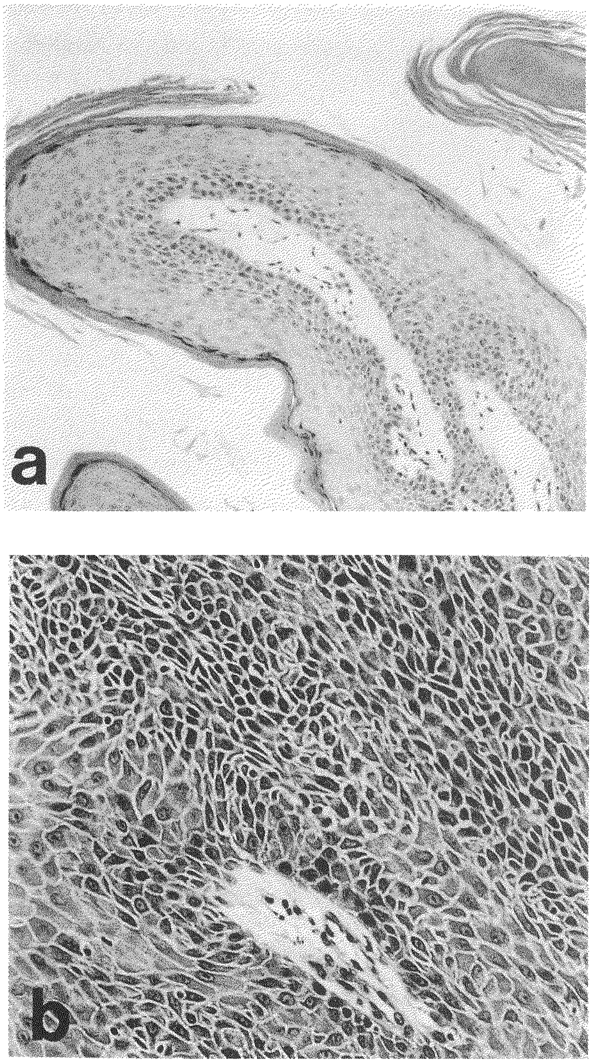

FIGURE 4 Histopathological findings of the lesion resected at the second operation. a: Papillary outgrowth of the stratified squamous epithelium with marked hyperkeratosis is observed (H.E. × 40). b: Dysplastic cellular changes with some mitoses and nuclear atypia in the nuclei are present in the basal cell layer, and the cells in the intermediate cell layer have enlarged (H.E. × 100).

microscopic laser surgery using a direct laryngoscope was performed under inhalation anesthesia by endotracheal intubation in November 1994. A hairy whitish lesion with exophytic outgrowth was observed extending from the center to slightly posterior of the membranous portion of the right vocal fold (Fig. 3a). A carbon dioxide laser was used, and while vaporizing the normal mucous membrane around the lesion with continuous mode of non-contact irradiation at a power output of 10W, the lesion was completely resected. The laser was also used to vaporize the deep layer of the vocalis muscle (Fig. 3b). Histopathological examination resulted in a diagnosis of verrucous carcinoma of the larynx. The patient was subsequently treated with oral UFT, 600mg (3×) a day as adjuvant chemotherapy on an outpatient basis. Local findings

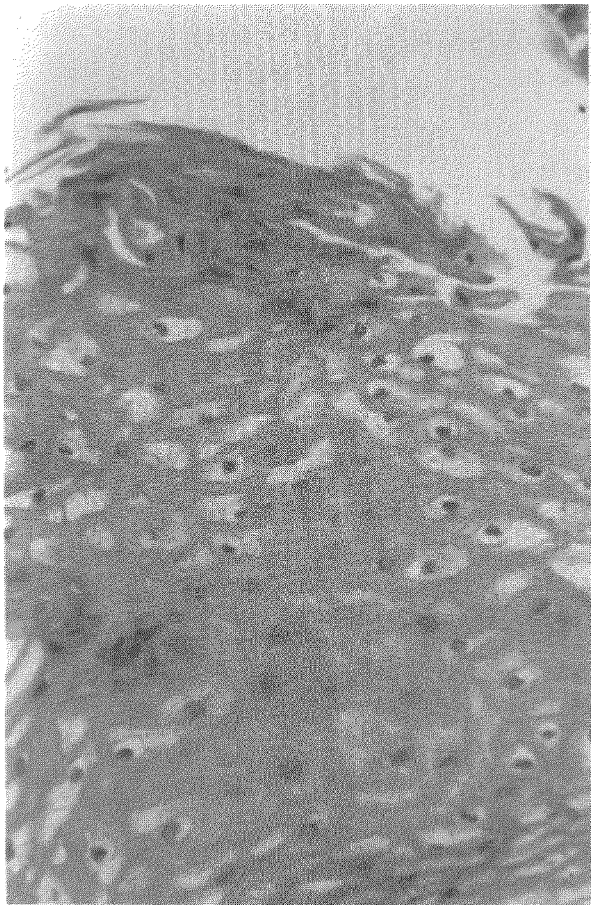

FIGURE 5 Koilicytosis. Evidence of koilicytosis comprised of prominent vacuolated cells with clear cytoplasm and dark pyknotic nuclei is observed from the superficial layer to the intermediate layer in a portion of the resected specimen (H.E. x 200).

twenty-one months after the operation showed complete disappearance of the lesion. There have been no local reccurence or evidence of metastasis.

### Histopathological Findings

The histopathological examination only revealed hyperkeratosis in the specimen excised at the first surgery. A specimen of the lesion resected at the second laser surgery was stained with hematoxylin and eosin. It showed papillomatous proliferation of the stratified squamous epithelium associated with marked hyperkeratosis (Fig. 4a). Dysplastic cellular changes with some nuclear mitoses and nuclear atypia were present in the basal cell layer, and the cells in the intermediate cell layer exhibited enlargement (Fig. 4b). Koilocytosis, consisting of prominent vacuolated cells with clear cytoplasm and pyknotic nuclei, was observed from the superficial layer to the intermediate layer in a portion of the specimen (Fig. 5). A definite diagnosis of laryngeal verrucous carcinoma was made histopathologically based on the above findings.

### DISCUSSION

The morphologically and biologically differentiating features of verrucous carcinoma with relatively low grade of malignancy that developed in the oral cavity were well defined by Ackerman, who coined the term "verrucous carcinoma" in 1984 [1]. As a result, it is also known by the name of "Ackerman's tumor". It is characterized as a whitish to grayish-white papillomatous or warty tumor. This slowly-developing neoplasm exhibits invasive down-growth into the deep mucosal layer, and it is reported to be most common in elderly men [1]. Verrucous carcinoma was reported in the larynx for the first time in 1966 [2]. It generally tends to develop in the oral cavity, and it has been reported that only approximately 10% of these neoplasms develop in the larynx [2]. Although this neoplasm has relatively low grade of malignancy, develops slowly and is characterized by a low rate of metastasis, it is defined as a distinct, histopathological and clinical variant of well-differentiated squamous cell carcinoma [3]. The reported incidence of verrucous carcinoma is approximately 1-4% of all malignant laryngeal neoplasms [3–6]. Its usual clinical appearance in the larynx is reported to be as a warty, pale whitish, bulky, exophytic outgrowth and sometimes appearing cauliflower-shaped [3]. These clinical characteristics of this neoplasm were also present in our case, but the tumor had a hairy external appearance, resembling a brush, more than being warty or bulky. Presenting as a hairy whitish tumor appears to be relatively uncommon. Verrucous carcinoma of the larynx is histopathologically characterized by being composed of islands and solid cords of very highly differentiated squamous epithelial cells, and its surface is covered by markedly hyperkeratotic cells [3]. Cytological and histological atypia is lacking or minimal, and mitoses are also rare. If they are present histopathologically, they tend to be concentrated in the basal-parabasal cell layer [3]. Thus, this neoplasm shows little evidence of malignancy, and can be regarded as having a low grade of malignancy [3]. Koilocytosis with prominent vacuolated cells containing dark pyknotic nuclei has occasionally been observed [7]. The second histopathological examination in our case revealed these distinctive histopathological features in the resected specimen. It seems to have been impossible to make a definitive diagnosis at the time of the initial histopathological examination because only the superficial keratotic lesion was excised with the forceps when the tissue specimen was obtained. It can be concluded that it is necessary to make a large en bloc excisional biopsy, including the deep layer of the mucous membrane, when removing the tissue specimen if this neoplasm is suspected.

Although the etiology of verrucous carcinoma of the larynx is unclear, the majority of the patients are cigarette smokers, and it seems to be closely associated with smoking [2,3]. Human papillomavirus (HPV) DNA has recently been detected in this neoplasm, and the possibility that infection by HPV induces its development has also been suggested [7,8,9,10]. In particular, results obtained with the polymerase chain reaction suggested an association between this neoplasm and HPV types 16 and/or 18 [9]. Although the tissue specimen obtained from our patient was not specifically tested for HPV, histopathological examination revealed koilocytosis in a portion of the specimen when stained with hematoxylin and eosin. Koilocytosis is a cytopathic effect of HPV infection characterized by the presence of prominent vacuolated cells possessing clear cytoplasm and pyknotic nuclei, and it is one of the histopathological findings frequently observed in laryngeal papillomatosis [11]. Thus, our case also appeared to have evidence of HPV infection.

Surgical treatment is usually recommended as the treatment of choice for verrucous carcinoma of the laryx [2–6]. If extention to cartilage, extralaryngeal spread or deep infiltration to the paraglottic space is observed, total laryngectomy may be selected when necessary [3]. However, if the lesion is localized unilaterally within laryngeal cavity, control by conservation surgery, such as cordectomy by laryngofissure and partial laryngectomy, is considered possible [3]. Endolaryngeal microscopic laser surgery has also been reported to be useful [12]. On the other hand, radiotherapy for this neoplasm is reported to be associated with a high rate of recurrence [3,5]. Conversion to anaplastic carcinoma has also been reported when treated with radiotherapy [2,4]. Although the efficacy of chemotherapy for this neoplasm is unknown, one patient who obtained a complete response to treatment with oral tegafur has been reported [13]. In our patient, the lesion was confined to the membranous portion of the right vocal fold. There was a recurrence because simple excision with forceps was performed at the first operation. In the second operation, however, endolaryngeal microscopic laser surgery using a direct laryngoscope was performed and followed by adjuvant chemotherapy with oral UFT. The patient's course has been favorable to date, 21 months postoperatively, with no evidence of recurrence or metastasis. Thus, endolaryngeal microscopic laser surgery plus postoperative adjuvant chemotherapy with oral UFT appears to be worth trying as the treatment of first choice for verrucous carcinoma of the larynx limited to one side.

### *References*

[1] Ackerman, L.V. (1948). Verrucous carcinoma of the oral cavity. *Surgery,* **23:** 670–678.

[2] Kraus, F.T., Prez-Mesa C. (1966). Verrucous carcinoma; Clinical and pathologic study 105 cases involving oral cavity, larynx and genitalia. *Cancer,* **19:** 26–38.

[3] Ferlito, A. (1933). Atypical forms of squamous cell carcinoma. In: Ferlito A, ed. Neoplasms of the larynx. New York: Churchill Livingstone, 135–167.

[4] van Nostrand, A.W.P., Olofsson, J. (1972). Verrucous carcinoma of the larynx: A clinical and pathologic study of 10 cases. *Cancer,* **30:** 691–792.

[5] Ferlito, A., Reacher, G. (1980). Ackerman's tumor (verrucous carcinoma) of the larynx: A clinicopathologic study of 77 cases. *Cancer,* **46:** 1617–1630.

[6] Biller, H.F., Bergman J.A. (1975). Verrucous carcinoma of the larynx. *Laryngoscope,* **85:** 1698–1700.

[7] Abramson, A.L., Brandsma, J., Steinberg B. *et al.* (1985). Verrucous carcinoma of the larynx: Possible human papilloma virus etiology. *Arch Otolaryngol,* **111:** 709–715.

[8] Shroyer, K.R., Greer, R.O., Fankhouser, C.A., *et al.* (1993). Detection of human papilloma virus DNA in oral verrucous carcinoma by polymerase chain reaction. *Mod Pathol* **6:** 669–672.

[9] Fliss, D.M., Noble-Topham, S.E., McLachlin, C.M. *et al.* (1994). Laryngeal verrucous carcinoma: A clinicopathologic study and detection of human papillomavirus using polymerase chain reaction. *Laryngoscope,* **104:** 146–152.

[10] Kasperbauser, J.L., O'Halloran G.L., Espy, M.J. *et al.* (1993). Polymerase chain reaction: Identification of human papilloma virus (HPV) DNA in verrucous carcinoma of the larynx. *Laryngoscope,* **103:** 416–420.

[11] Abramson, A.L., Steinberg, B., Winkler, B. (1987). Laryngeal papillomatosis: Clinical, histopathologic and molecular studies. *Laryngoscope,* **97:** 678–685.

[12] Milford, C.A., O'Flynn, P.E. (1991). Management of verrucous carcinoma of the larynx. *Clin Otolaryngol,* **16:** 160–162.

[13] Kitano, H., Kitajima, K. (1994). Laryngeal verrucous carcinoma: Effective treatment with tegafur. Auris • Nasus • Larynx(Tokyo) **21:** 64–68.

